# O039. Case-control genetic association studies in migraine: a 7-year experience at the Interinstitutional Multidisciplinary Biobank (BioBIM) of IRCCS San Raffaele Pisana

**DOI:** 10.1186/1129-2377-16-S1-A120

**Published:** 2015-09-28

**Authors:** Piero Barbanti, Raffaele Palmirotta, Maria Laura De Marchis, Cristiano Ialongo, Gabriella Egeo, Cinzia Aurilia, Luisa Fofi, Serena Piroso, Federica Fratangeli, Maria Giovanna Valente, Fiorella Guadagni

**Affiliations:** Headache and Pain Unit, Department of Neurological Motor and Sensorial Sciences, IRCCS San Raffaele Pisana, Rome, Italy; Interinstitutional Multidisciplinary Biobank (BioBIM) Biomarker Discovery and Advanced Technologies (BioDAT), IRCCS San Raffaele Pisana, Rome, Italy; Telematic University San Raffaele, Rome, Italy; Department of Internal Medicine, Tor Vergata University, Roma, Italy; Fondazione San Raffaele, Ceglie Messapica, Italy

## Background

Current advances in molecular biology, together with the development of Biobanks as stable sources of biologic material, are enhancing the possibility of detecting genetic factors involved in the molecular pathogenic mechanisms of migraine, a complex neurological disorder classified as the seventh most disabling disease worldwide.

## Results

To date, the migraine section of the Interinstitutional Multidisciplinary Biobank (BioBIM) of IRCCS San Raffaele Pisana has recruited 863 migraine patients and 400 healthy individuals as controls. Each biological sample has been associated with extremely detailed socio-demographic and clinical features of the donor[1]. Thanks to this extended sampling, our group was able to identify significant correlations between several genetic variants and specific migraine features. In a study carried out on the V129M polymorphism of the prion protein gene (PRNP) we showed an association between the 129VV genotype and an earlier age at migraine onset[2]. By investigating the common I/D polymorphism of the angiotensin I-converting enzyme (ACE) gene we found that the I/I genotype (associated with reduced ACE and angiotensin II serum levels, hence to reduced glutamatergic and increased GABAergic neurotransmission) seems to confer a milder migraine phenotype in patients with migraine with aura and chronic migraine[3]. Focusing on the role played by factors controlling oxidative mechanisms in the pathophysiology of migraine we described a striking correlation between the rs4880 variant of the superoxide-dismutase 2 (SOD2) gene (associated with reduced antioxidant activity) and the presence of unilateral cranial autonomic symptoms in patients affected by migraine with aura[4]. Given the strong influence of female gender and sex female hormones on migraine susceptibility, we also investigated the possible association of the rs1042838 polymorphism of Progesterone receptor gene (PGR) with this disease. Indeed, our data highlighted a linear relationship between the copy number of the T allele and the age of migraine onset[5]. Finally, we excluded any correlation between polymorphisms rs4818 and rs4680 of Catechol-O-Methyltransferase (COMT) gene and migraine, suggesting to look over COMT to explain catecholamine derangement in migraine, exploring enzymes involved in catecholamines synthesis and catabolism such as monoamine-oxidase, dopamine beta hydroxylase, tyrosine hydroxylase or tyrosine decarboxylase[6].

## Conclusions

Our Biobank dedicated to migraine has proven to be a valuable resource to conduct molecular studies on this disease, allowing the identification of a new potential biomarker for detection of asymptomatic individuals at increased risk for migraine development, in addition to providing the basis for the design of more tailored and effective therapies.

## Conflicting interests

None.
